# Measuring position sense

**DOI:** 10.1113/EP092190

**Published:** 2024-11-22

**Authors:** Uwe Proske

**Affiliations:** ^1^ School of Biomedical Sciences Monash University Clayton Victoria Australia

**Keywords:** antagonist muscle, muscle spindle, position sense, thixotropy, voluntary contraction

## Abstract

Position sense is arguably more important than any of the other proprioceptive senses, because it provides us with information about the position of our body and limbs in relationship to one another and to our surroundings; it has been considered to contribute to our self‐awareness. There is currently no consensus over the best method of measuring position sense. We have recently measured position sense with three commonly used methods. These were two‐arm matching, one‐arm pointing and one‐arm repositioning, all carried out by blindfolded subjects with their lightly loaded forearms moving in the sagittal plane. It is currently believed that muscle spindles are the principal position sensors. We posed the question, was there evidence for spindles participating in the generation of position sense with each method? The indicator of spindle activity we used was the presence of thixotropic errors in the position signal, in response to conditioning voluntary contractions of forearm muscles. Based on this criterion, there was evidence of spindles contributing to position sense with all three methods. It was concluded that the spindle contribution to the position signal and the extent to which this was processed centrally was different with each method. It is argued that a case could be made for the existence of more than one position sense. Differences between the methods have implications for their meaning in a clinical setting.

## Abstract (highlights)

- **What is the topic of this review?**
  This mini‐review discusses methods of measuring position sense at the human forearm.
- **What advances does it highlight?**
  Three classes of methods were considered: position sense by two‐arm matching, one‐arm pointing and one‐arm repositioning. A contribution from muscle spindles to generation of the position sense signal could be detected by means of voluntary conditioning contractions of elbow muscles. For all three methods, evidence was obtained of spindles contributing to the generation of position sense, but to different extents. These differences in the processing of the spindle signals have led to the suggestion of the existence of more than one position sense.

## 1 INTRODUCTION

Almost every week, a new publication on the subject of proprioception appears in the literature, and it often includes studies of position sense. The topic is so popular because it is interesting and important. It provides useful background information for certain clinical conditions and plays a central role in sensorimotor control. Furthermore, given that position sense is believed to contribute to our self‐awareness (Cole, 1995, 2016), the subject promises to tell us something about ourselves, and we are always interested in that! Unfortunately, when it comes to measuring position sense, there is no consensus view about the best method to adopt. Often the most convenient, rather than the most suitable, is chosen, and that risks obtaining contradictory outcomes. This mini‐review is a plea to reassess methods of measuring position sense and to accept that the subject is more complex than we might have thought, with different methods leading to different outcomes.

The sense of limb position allows us to know, at any point in time, where different parts of our body are, in relationship to each other and to our surroundings. Furthermore, position sense plays an important role in motor control, specifically in reaching movements (Desmurget et al., 1995; Sarlegna & Sainburg, 2009). Indeed, there is evidence that disturbance of position sense with muscle vibration, before onset of a reaching movement, interferes with movement accuracy (Larish et al., 1984).

The question debated here is, how should position sense be measured? Different methods of measurement of proprioception have been reviewed before (Han et al., 2016; Horvath et al., 2023), but they considered aspects of proprioception in addition to the sense of position. In particular, they included the sense of movement. The sense of movement is distinct from the sense of position (McCloskey, 1973a) and is believed to be generated by the primary endings of spindles, while both primary and secondary endings contribute to position sense (Banks et al., 2021). Therefore, although both senses contribute to proprioception, each measures something different. Although that might not be of any consequence in some clinical settings, interpreting the measurements can be problematical. Likewise, the classical clinical test of proprioception performed by the neurologist is to waggle the subject's big toe up and down, asking them whether they felt it move and, if so, in what direction. This also tests both the senses of movement and of position.

A few more words about movement sense. It is distinct from position sense, being generated during movement of a muscle from one length to another, whereas position sense derives from the maintained level of activity at a given length. Several arguments have been made in support of the view that position and movement are two distinct senses and that the centrally projected afferent information for each sense is processed separately. For a detailed discussion, see Proske and Gandevia (2012). In the present review, movement sense will not be discussed further.

The choice of a method of measurement of position sense depends, of course, on what purpose it is to serve. However, if it invokes several mechanisms at the same time, the outcome is likely to change depending on the conditions. We have preferred to limit ourselves to measurements of position sense because it is believed to arise, specifically, from maintained levels of activity in muscle spindles, although it has been claimed that, in some conditions, signals from skin and joint receptors might contribute (for details, see Proske, 2023).

In a recent account (Roach et al., 2023), three commonly used methods of measuring position sense were described. The way we chose to study each method was to use a property, unique to spindles as contractile sensory receptors, called thixotropy. Thixotropic conditioning allows the generation of controlled, predictable changes in spindle responsiveness. With each of the three methods of measurement, we looked for evidence of a pattern of position errors characteristic of thixotropy which would, therefore, confirm a contribution from spindles to the measured value. Therefore, the underlying question was, did spindles contribute to all commonly used methods of measuring position sense and, if so, to what extent?

Thixotropy is a property unique to all striated muscle, including the intrafusal fibres of muscle spindles (Proske et al., 1993, 2014). In a resting muscle, a small number of stable cross‐bridges form between actin and myosin filaments in sarcomeres. These stable bridges begin to form immediately after a contraction. If the muscle is then stretched, the stable bridges detach to re‐form at a longer length. When a muscle is held at a long length, stable bridges will have formed at that length. If the muscle is then shortened, the stable bridges do not immediately detach, as a result of the compressive forces during shortening, and they act as a splint on muscle fibres, which are therefore unable to shorten incrementally. This leads muscle fibres to fall slack. The resulting low passive tension in slack intrafusal fibres reduces the maintained strain on spindle sensory endings and lowers spindle responsiveness to changes in length. Therefore, depending on whether measurements are made after a contraction or after a stretch, the accompanying spindle sensitivity will be different. This provides the opportunity for manipulating spindle sensitivity in experiments.

In view of the broad title of this mini‐review, we considered discussing as many as possible of the different methods of measurement encountered in the literature. However, we included one important reservation. Our earlier work had shown that if muscles had not been conditioned by stretch or contraction before a measurement, to put them into a defined thixotropic state, potentially large, uncontrolled errors would creep into the measurements (Gregory et al., 1988). Doing nothing was not an option, because it meant that the values measured lay on an uncertain baseline. Therefore, errors measured with methods that did not include some form of muscle conditioning were treated with caution.

As a starting point, we considered the classical method first used to establish a role for muscle spindles in position sense (Goodwin et al., 1972). This was the two‐arm matching method. These authors postulated that muscle spindles in forearm muscles of both arms contributed to the generation of position sense (for a review, see Proske and Chen, 2021). We then asked the question, how good are subjects in detecting limb position when spindle signals from only one arm are available? This led us to the method of measuring position sense by pointing. Here, the subject was required to point, with one arm, to the perceived position of the other arm, hidden behind a screen. With this method, it was important to make sure that there was no unintended contribution from proprioceptors of the arm doing the pointing and, in the process, introducing elements of two‐arm matching into the measurement. Examples of studies using the method of pointing include Velay et al. (1989), Longo and Haggard (2010), Ingram et al. (2019) and Darling et al. (2024).

Finally, by far the commonest method used to measure position sense, especially in circumstances where the results have important clinical implications, is the method of repositioning, reviewed by Goble (2010). Some of the variants of the method are described below. In the application of this method, it is often tacitly assumed that the receptors responsible for position sense by repositioning are the muscle spindles, although no evidence for such a conclusion has ever been provided. On some occasions, the results of measurements made in repositioning are directly compared with matching, without any mention of possible differences in underlying mechanisms (Elangovan et al., 2014). All of this has led to uncertainty and a lack of consensus over the sizes of position errors. A redeeming feature of the repositioning method is that the impact of thixotropic effects on measured errors is minimal. Here, the interpretation is put forward that processing of repositioning signals largely takes place centrally, independently of any changes in peripheral afferent discharges.

These three methods have been chosen as representative of the wide range of methods encountered in the literature. In selecting the conditions of their experiments, Roach et al. (2023) chose to move the arms in the sagittal plane, with the subject holding their arm unsupported, providing the opportunity for the force of gravity to exert an influence (Soechting, 1982), and subjects were always blindfolded. Therefore, these were strictly proprioceptive tasks, and the important influence of vision was excluded. We had, of course, not excluded vision entirely, given that visual information about the location of the arm in space, acquired before the blindfold was applied, was likely to exert an influence. By simplifying the conditions of the experiments in this way, we hoped to achieve a better understanding of the underlying processes.

The outcome of the experiments described by Roach et al. (2023) showed that there was evidence of spindles participating in position sense measured with all three methods. However, the sizes of the thixotropic errors were very different. It led to the conclusion that the amount of central processing to which the spindle signal was subjected was rather different. Although Roach et al. (2023) recognized the importance of their findings, they did not take the extra step to realize, more broadly, that it was likely that distinct mechanisms were involved with each method and that the widely held view of position sense as a single entity was no longer tenable. Consideration should now be given to the implications for somatosensation and movement control of having three distinct position senses.

The observations described here were all made at the elbow joint. However, for position sense by two‐limb matching, similar studies had been carried out at the wrist (Walsh et al., 2013), at the knee (Givoni et al., 2007) and at the ankle (Vuillerme et al., 2006). It is our impression that, at least for matching, the evidence supports the view that similar mechanisms are operating at different joints, when the task is to align two corresponding parts of the body (Proske & Chen, 2021). We therefore think it is safe to generalize our findings to most joints.

## 2 TWO‐ARM MATCHING

**FIGURE 1:**
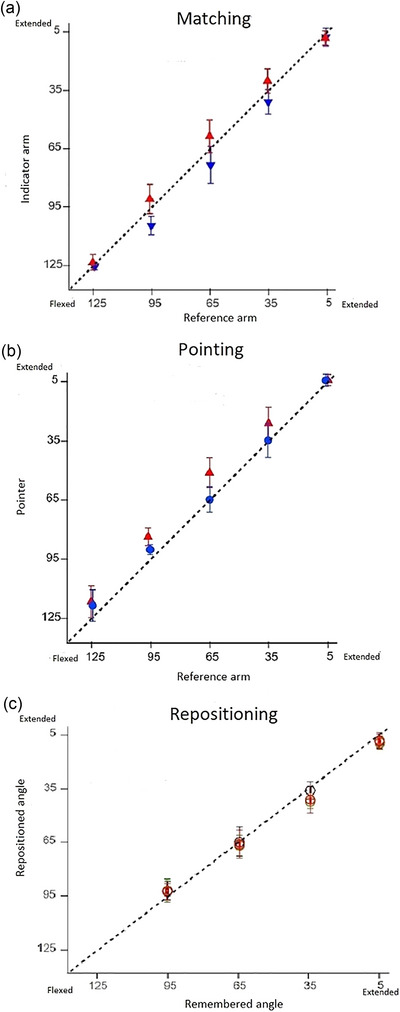
Position sense, measured over the full working range of the human forearm, by two‐arm matching (a), by one‐arm pointing (b) and by repositioning (c). (a) One of the blindfolded subject's forearms, the reference, was conditioned with a half‐maximum voluntary contraction of elbow muscles at 125° (forearm flexed) and extended to a randomly selected test angle; the indicator was conditioned at 5° (forearm extended), then moved towards the reference to a matching position (red triangles). Then the sequence was reversed, with the reference conditioned at 5° and the indicator at 125° (blue triangles). Matching angles are shown as means (±SD) for three repetitions by each subject, pooled for 11 subjects, measured over the full range of elbow angles, 125°–5°. Dashed line, line of equality, the position of the indicator if it had accurately matched the reference. (b) Position sense by pointing. The position of the hidden forearm is indicated with a pointer moved by the other arm. The elbow muscles of the hidden arm were conditioned at 125° (red triangles) or 5° (blue circles), then the arm was moved to the test angle, where its position was indicated with the pointer. Values shown as means (±SD) for the two conditionings, for three repetitions, for 11 subjects, over the full range of elbow angles. Dashed line, zero error. (c) Position sense was measured by repositioning. From a starting position of 125° the forearm was moved into extension to one of four randomly presented test angles (95°, 65°, 35° and 5°), and the subject was asked to remember that angle. After returning to the starting position, they were asked to reproduce the remembered position. This sequence was repeated, but with a starting angle of 5°. Values are shown as means (±SD) for three repetitions for each of 11 subjects. Black open circles, arm muscles left unconditioned. Green open circles, arm muscles conditioned at 125° after the learning stage, before the reproduction stage. Red open circles, arm muscles conditioned before the both learning and reproduction stages. Dashed line, zero error. Figures redrawn from Roach et al. (2023).

The largest thixotropic errors were seen in two‐arm matching (Figure 1a). We assumed that it meant the method involved less central processing than the other two, and this allowed the spindle signal to dominate the outcome. In earlier experiments on two‐arm matching at the forearm, using thixotropic conditioning, it was shown that as subjects aligned their arms, the final position of the indicator arm depended on the difference in signal coming from spindles in the two antagonist elbow muscles of each arm, in addition to the difference in signal between the two arms (Proske & Chen, 2021; Proske & Gandevia, 2018).

To spell this out in more detail, thixotropy makes use of muscle conditioning as a means of changing spindle discharge levels without changing muscle length. Here, before matching the arms, the subject conditioned them with a voluntary contraction at opposite ends of the working range of the forearm (125° and 5°) to maximize thixotropic errors. As a result of its conditioning in a flexed position (125°), the reference arm, sitting at the test angle, had a higher level of flexor discharge than expected for that angle, leading it to be perceived as more extended than was really the case. Likewise, the indicator, after conditioning at 5°, had a higher level of extensor activity and therefore felt more flexed. During the match, these errors add, and the indicator adopts a more extended matching position (red triangles, above the line of equality, Figure 1a). When conditioning is reversed, the reference extension conditioned and the indicator flexion conditioned, the errors are in the opposite direction; the reference is perceived as more flexed than is really the case and the indicator feels more extended, leading to errors into flexion (blue triangles, below the line of equality, Figure 1a). This leads to a total swing of 12° of error in the mid‐range of elbow angles. We believe these are the maximum errors achievable with the conditioning method, under the conditions of this experiment.

During the process of generating these errors, the subject was confident of having achieved an accurate match, and they were unaware of the mismatch between their arms. It seems the brain has access to a calibrated spindle discharge–muscle length–joint angle relationship (Held & Bauer, 1967). If, as a result of muscle conditioning, the prevailing discharge coming from forearm muscles at a particular length is higher than the calibrated value, this is interpreted as a longer muscle than is really the case, a more flexed or extended joint. This misperception leads to errors in matching positions.

When the reference arm is sitting at the test angle ready to be matched, and the indicator is moved by the subject to a matching position, do the movement signals generated in indicator spindles and, perhaps, also a centrally generated effort signal, contribute to matching performance? The answer for movement is, ‘yes’. Moving the indicator over a range of angles during the match generates a small, movement‐related error in the final adopted position. For movements at the elbow over 40°, the error was 5° (Chen et al., 2012: fig. 3). Concerning effort sensations, we have, in the past, measured position sense in a matching task, comparing position errors when the reference arm was unloaded and when it supported a load that represented 25% of maximum contraction force. Despite the greater effort required to support the loaded arm, this did not alter position sense accuracy (Allen et al., 2007).

Instead of plotting the position of the indicator arm against that of the reference, as in Figure 1a, the real errors for a given test angle can be measured. When plotted against elbow angle, this yielded a bell‐shaped curve for the five test angles, directed upward, in the direction of elbow extension, when the reference was conditioned at 125°, and a similar curve directed downward, in the direction of elbow flexion, when the reference was conditioned at 5°. A feature was the symmetrical, mirror shape of the curves (Roach et al., 2023: fig. 3a). We propose that these curves are determined by the rates of spindle discharges coming from the two arms during the matching process. For the three mid‐range angles, 95°, 65° and 35°, with the arms approaching each other from opposite directions, the combined error signals have changing values, peaking at 65° and falling at 95° and 35°. We suggest that this is the result of muscle length‐dependent changes in spindle signals in the two arms, as a result of small alterations in the effects of conditioning. For the extreme angles, 125° and 5°, during the match one arm did not move and therefore high spindle rates were compared in both arms, hence it yielded small errors. However, in saying that, we have not excluded the possibility of an influence of joint receptors acting to reduce position errors at extreme matching angles (Proske, 2024).

If we are right, and the curved relationship for length dependence of matching errors is attributable to changing spindle rates, it implies a direct transformation of spindle signals into position errors. It reinforces the view that a minimum of central processing is involved. It was recently shown in human subjects lacking spindles in limb muscles that position sense at the forearm was normal. It was proposed that the role of spindles had been taken over by skin receptors (Smith et al., 2020). Perhaps such flexibility in the interchange between afferent signals from different sources is possible because of the simple, direct conversion of the afferent information into a sense of joint position.

## 3 ONE‐ARM POINTING

In pointing, the subject uses a pointer to indicate the position of an arm hidden behind a screen. The most important consideration for one‐arm pointing is that it involves signals from only one arm. Therefore, the mechanism for locating the position of the hidden arm must be different from that in two‐arm matching. The evidence that signals from the arm doing the pointing do not contribute to the measurement is based on the observation that if the subject indicates the position of the hidden arm verbally, without moving their pointing arm, the outcome is much the same (Chen et al., 2021). In a naive view, it might have been thought that after muscle conditioning the errors in one‐arm pointing would be half as big as in two‐arm matching, because now signals are coming from only one arm. But, unfortunately, it is not as simple as that, and we noted two features unique to position sense measured by pointing.

The first was that in pointing, position sense errors for mid‐range angles were all shifted in the direction of extension of the real position of the arm (all values, triangles and circles, lay on or above the line of equality, Figure 1b). This confirmed what we had seen before (Chen et al., 2021). We do not know why such a shift occurred. Our provisional explanation is that, at the elbow, the flexor spindle signal is stronger than the extensor signal because flexor muscles are larger and contain 20% more spindles than the extensors (Voss, 1971). The flexor population response is therefore larger, which is interpreted by the brain as a signal bias in the direction of extension. The second feature was that this shift was 8°–10° over the mid‐range of elbow angles, 85°–45°. However, when measurements were made on a more extended arm, the errors became smaller, to lie close to zero at a test angle of 5° (Chen et al., 2021: fig. 5). This finding was interpreted as the spindle signal coming under the influence of joint receptors as the arm got closer to the limit of its working range (Proske, 2023, 2024).

Inspection of the distribution of errors in the pointing task (Roach et al., 2023: fig. 4b) showed that after conditioning of arm muscles at 125°, when flexor activity was expected to be dominating, errors were distributed in a bell‐shaped relationship, as had been seen in two‐arm matching. However, error values lay further in the direction of extension than seen in matching, although now the signals were coming from only one arm. After conditioning at 5°, when extensor activity would be expected to be prominent, directing errors into flexion, there was no sign of an inverted curve as seen in matching, and error values all lay close to zero or a little above it. Given that error values after conditioning at 125° all lay above those after conditioning at 5°, some aspects of thixotropic influences were still apparent. However, the reciprocal distribution of errors seen in two‐arm matching had been lost. This led to the important conclusion that the expected thixotropic pattern of errors had not been fully preserved in pointing and that other influences were exerting an effect. This will be the subject of future experiments.

Additional observations about pointing were made by Velay et al. (1989). In a comparison between matching and pointing, before making the measurements, subjects underwent a brief training period wearing prism lenses that shifted the visual field by 11°. After they were blindfolded and the experiment was begun, subjects made new, significant errors in the pointing task, but not in the matching task. This suggested that before the pointing, subjects took note of the spatial orientation of their arm relative to its surroundings, and this information was used during the pointing. Presumably, during a trial, the spatial information was remembered and used in combination with spindle signals coming from the hidden arm at the test angle to determine the position of the arm. Velay et al. (1989) mentioned that the time elapsing between placement of the target arm and pointing to it impaired pointing accuracy, but that this was not the case for matching. It implies that with pointing, the acquired spatial memory fades with time.

## 4 REPOSITIONING

Repositioning is, arguably, the most important method of measuring position sense, because it is frequently the method of choice in clinically relevant studies. The blindfolded subject has one arm placed at a test angle; they are asked to remember that angle and then, several seconds later, to reproduce it. Using only one arm is the simplest version of the method, and there are numerous other variants. For example, the subject is asked to remember a test angle with one arm, and then, after withdrawing the arm, they reproduce the angle with their other arm (‘contralateral remembered’; Goble, 2010). This is considered a more challenging task because it is thought to involve inter‐hemisphere transfer in addition to memory. Another version is, ‘Which of these two remembered positions is closer to the body?’ (Elangovan et al., 2014). Alternatively, a test angle is presented to the subject and the question is, ‘Which of three previously learned positions is closest to the test angle?’ (Shihoyara et al., 2023). There are other versions, and they all have in common a memorizing stage.

The way we did our experiment was to carry out a one‐arm repositioning trial with an unconditioned arm and then to repeat the trial, but after elbow flexor and extensor muscles had been conditioned with a contraction. We argued that once the position of the test angle had been learned, based on the prevailing spindle signal, if elbow muscles were then conditioned and this altered spindle discharge rates, this would interfere with recall of the remembered signal, and errors would arise. In the event, it was found that in repositioning, the influence of conditioning on position errors was less than for either matching or pointing. The reproduced values lay close to the remembered angles, and it was not immediately obvious that conditioning had disturbed position sense at all. All values lay on top of one another, making it difficult to distinguish the three conditions (Figure 1c).

Did that mean spindles did not contribute to the generation of position sense by repositioning? However, subsequent statistical analysis showed that for flexor conditioning, but not for extensor conditioning, there had been a significant effect of the conditioning contraction on repositioning errors. Here, it occurred to us that the bias observed with position values in one‐arm pointing might also be present in repositioning; the stronger signal with flexor conditioning pushing values to statistical significance. However, the important point is that although there might have been overall statistical significance, differences were too small to reach significance in comparisons between individual angles. In addition, closer scrutiny of the errors showed that they did not follow meaningful length‐dependent changes as had the errors for matching and pointing (Roach et al., 2023: fig. 6). Clearly, the mechanism underlying repositioning was substantially different from that for the other two methods of measuring position sense.

We have attempted to compare the sizes of the errors obtained with each of the three methods. It is not straightforward to make such a comparison. In two‐arm matching, signals from muscles of both arms contribute; in pointing only one arm was involved, and in repositioning it was a recall error. The most reliable comparison can be made after conditioning of arm muscles at 125°. The average error value over the three mid‐range angles (35°, 65° and 95°) was +5.9° for matching, (range +5.4° to +6.8°, positive value, error into extension), +11.9° for pointing (range +9.1° to +14.1°) and +0.5° for repositioning (range −1.7° to +3.3°; Roach et al., 2023). Such a comparison emphasizes the large errors into extension in pointing and the small errors in repositioning.

## 5 DISCUSSION

Our recent experiments were based on the question, were the different methods of measuring human position sense sufficiently similar to allow for a direct comparison between their respective values? Alternatively, was the outcome of the three methods sufficiently different to make it necessary to treat each one separately? In our conclusions, we were more inclined to the latter view, that is, interpretation of errors measured with each method was likely to involve substantial differences in meaning. Such a conclusion has important implications for inferences drawn from position sense measurements made in a clinical setting.

But it does more than that! It is emerging that the term position sense represents a collection of senses, each with its own characteristics, probably served by unique underlying mechanisms and designed to serve specific purposes in sensorimotor control. Our understanding of proprioception is broadening, and we are beginning to appreciate that interpreting what, at first sight, appeared to be a relatively straightforward measurement, is becoming more complicated.

In a comparison between two‐arm matching and one‐arm pointing, Velay et al. (1989) concluded, ‘…these two tasks do not test the same position sense’. For matching, they suggested that forearm position was represented centrally in terms of relative angular positions in intrapersonal space. Such a process would not be expected to be affected by prismatic distortions of the visual field, presented to the subject before carrying out a two‐arm matching task. However, they pointed out that the matching mechanism would not permit location of limb extremities in space. A different form of coding would be necessary, one that required continuous knowledge of all parts of the limb in extrapersonal space. This was provided in position sense by pointing. It is vulnerable to distortions of the visual field presented before the task because it relies on position information for the arm in relationship to its external surroundings.

Lesions of parietal cortex often leave both the senses of matching and pointing disturbed. However, some subjects have great difficulty in locating one of their hands in space but are still able to carry out a simple two‐arm matching task (Velay et al., 1989). McCloskey (1973b) studied two split‐brain patients who had undergone section of their corpus callosum. When he studied their position sense in a two‐arm matching task, both subjects were found to be normal, and they also reported normal illusions in response to vibration of elbow flexors or extensors. McCloskey concluded that commissural connections below the neocortex were responsible for the normal behaviour of the two subjects. However, given that they had difficulty in locating and pointing to one hand with the other, it suggests that here the necessary information might not be transferable if the neocortical commissures were divided. These findings suggest that distinct central pathways are likely to be involved in position sense measured in matching and pointing.

In subjects with a sensory and autonomic neuropathy (HSAN III), functional muscle spindles are absent in both upper and lower limbs (Macefield et al., 2024). The neuropathy patients exhibited an ataxic gait, and the degree of gait impairment was correlated with the loss of position sense at the knee. Although lacking spindles, these subjects have normal skin receptors. Position sense at the knee, measured in a two‐limb matching task, showed that the poor matching performance could be improved by taping the knee (Macefield et al., 2016). It was suggested that the tape increased tensile strain in skin about the knee, and this led to recruitment of more skin receptors during the matching process, suggesting that the remnant position sense in these subjects was provided by receptors of cutaneous origin. Interestingly, position sense measured by matching of the forearms was found to be normal, and it was proposed that here the role of spindles had been taken over completely by skin receptors (Smith et al., 2020).

However, in all these subjects there was a deficit, typical of HSAN III sufferers, of a poor performance in the finger‐to‐nose pointing test. It suggests that at the forearm the role of spindles in two‐arm matching can be taken over by skin receptors, but not the role in pointing. If the proposal of Velay et al. (1989) is accepted, and in a two‐arm matching task the central representation of position sense is in terms of relative angular positions in intrapersonal space, it is conceivable that for this role a spindle signal can be replaced by signals of skin receptors. However, in a pointing task, sufferers of HSAN III seem not to be able to determine the location of their limb extremities. The afferent coding required for the acquisition of knowledge of all parts of the limb (or nose) in extrapersonal space, presumably provided normally by spindles, seems to be missing.

Therefore, this is an example of where interpretation of the findings is based on the method of measurement. It again hints at a fundamental difference between matching and pointing. Furthermore, it implies that for matching the afferent basis for position sense is not necessarily dependent only on spindles, unlike that for pointing. In contrast, for pointing a spindle signal is required to communicate between the sensory periphery and central, stored sites of information about different body parts and their location in extrapersonal space. If so, this represents a unique role for spindles in proprioception.

In an earlier experiment (Chen et al., 2021), we measured position sense by pointing, but instead of asking subjects to point with their other arm to the perceived position of the hidden arm, we asked them to select one of a series of trajectory lines drawn on the screen hiding the reference arm, and they reported verbally which line they thought lay closest to the felt position of the hidden arm. Subjects were able to indicate the position of the forearm just as accurately as when they had pointed to it with their other arm. This suggested that subjects had a spatial memory that could be converted to a visual frame of reference to guide the pointing arm or, conversely, a visual signal (slope of the line) could be converted into a proprioceptive one. If split‐brain subjects could not point with one hand to the other (McCloskey, 1973b), it suggests that they were unable to generate such spatial memories. It seems that they had difficulty in finding their hands in a similar manner to the HSAN III subjects trying to point to their nose. Perhaps, for spatial localization, not only are spindles required, but maybe their input to central sites from different parts of the body is necessary?

Borchers et al. (2011) reported a subject who had experienced a small, confined lesion of the hand area in the right postcentral gyrus that resulted in a proprioceptive deficit without any accompanying motor impairment. The lesion affected position sense by pointing, in both hands, suggesting an integration of contralateral and ipsilateral proprioceptive information already at this early processing stage, presumably mediated by callosal connections. Such observations underline the importance of bilateral inputs for acquisition of spatial information in pointing.

The distribution of errors in position sense measured by repositioning provided the most unexpected result of our study. There was a near absence of thixotropic effects and therefore, presumably, little contribution from muscle spindles to the remembered signal. What position sense by repositioning reveals is that we have a remarkably acute sense of spatial memory; it is laid down, with little influence from ongoing changes in peripheral afferent activity.

It has been reported (see Velay et al., 1989: p. 179) that in a repositioning task, if the subject was exposed to visual distortions by prismatic exposure after they had learned the position of the test angle, this did not interfere with the accuracy of repositioning. This result is consistent with the findings by Roach et al. (2023) that muscle conditioning after the learning stage had little effect on repositioning accuracy (Figure 1c). Our original hypothesis had been that in a repositioning task, during the learning stage, spindle signals coming from the arm participated in identifying the position to be remembered, and during repositioning they helped to guide the arm to the remembered position. We now know that is not correct, because thixotropic conditioning given before the repositioning stage had so little effect on the outcome. This finding has led us to doubt a major involvement of ongoing spindle activity in position sense by repositioning.

Our present hypothesis is that when a subject is asked to remember a test angle, the instruction triggers a process of identifying a central storage site for spatial information congruent with the test position. Selection of the site does not involve any direct reference to ongoing spindle activity in arm muscles and is entirely central in origin. A memory is established, which is then recovered during the subsequent repositioning stage. In the future, we want to test this idea further by generating thixotropic disturbances both before the memorizing stage and before the reproduction stage. If there were still no thixotropic influence on the position errors, this would support our view of an exclusively central origin of the errors.

The idea of the availability of highly specific, central storage sites for spatial information has led us to reassess some of our earlier observations. As mentioned above, the error distribution in position sense by pointing can be reproduced without moving the pointing arm, by verbally selecting one of a series of trajectory lines on the screen hiding the arm (Chen et al., 2021). We had assumed that the subject was converting the lines into different possible arm positions, involving a change in the frame of reference. However, it is conceivable that when the subject selected a line they were, in fact, accessing central stores of spatial information. That is, the subject was not using a pointing mechanism at all, but one related to position sense measured by repositioning. It is possible that something similar takes place in conventional pointing, although here the presence of sizeable thixotropic errors suggests that there is also a direct participation by ongoing spindle activity.

Which, then, is the best of the three methods to choose for measurements of position sense? The repositioning method has the advantage of simplicity of application and a relative insensitivity to thixotropy. It does, however, have a large memory component. Therefore, before making any measurements, subjects should first be screened for their memory retention capacity. Position sense by pointing provides information about position of the limb in extrapersonal space and, in that respect, is distinct from the other methods. It will be influenced by vision, and it has been shown to represent the positional information centrally in the form of a body model (Longo & Haggard, 2010). Perhaps the most straightforward method is measuring position sense using two‐limb matching. It is believed not to involve a memory component (Tsay et al., 2014) but is strongly influenced by spindle thixotropy, meaning that for accurate measurements of position sense, limb muscles must always first be conditioned. To summarize, each of the three methods has its own advantages and disadvantages. If these are taken into consideration, then all three will provide similarly accurate measures of position sense.

## 6 CONCLUSIONS

Our approach to the study of position sense has been a somewhat unorthodox one; we have not simply picked a commonly used method of measurement, then applied it to our experimental situation. Instead, we have begun by studying the sensory receptors themselves, believed to be the source of the position signal, then attempted to trace signs of their activity with different established methods of measurement. What that has revealed is that each method involves astonishingly different degrees of processing of the afferent signals, to the point that it could be said there exists more than one distinct position sense, each with its own role in sensorimotor control. It follows that the central destinations for the signals of muscle spindles for each of these senses are likely to be different. This brings with it the realization that proprioceptive sensations are more complex than we might have thought and that any inferences we draw from a chosen method of measurement are likely to be unique to that method and not transferable to other methods. This, of course, has important implications for clinical diagnostic studies of position sense in addition to opening a window to a daunting array of mechanisms.

The present review has led to three significant conclusions. First, there are probably at least three distinct position senses. Second, the information provided, based on the clinical circumstances of patients who lack muscle spindles in their limb muscles, has led us to propose that this leads to a disturbance of position sense measured by pointing, but appears not to affect some matching. For pointing, it suggests a role for spindles that goes beyond signalling muscle length changes; they are able to access spatial information that is necessary for position sense by pointing. Third, the absence of thixotropic errors in position sense by repositioning has led to the proposal that ongoing activity of peripheral receptors does not seem to be involved in the generation of the memory trace created when a subject is asked to remember a particular limb position. That conclusion makes position sense by repositioning unique; a sense dependent on short‐term memories of spatial information acquired simply by focusing attention on the present position of the limb, to be reproduced with astonishing accuracy.

## AUTHOR CONTRIBUTIONS

Sole author.

## CONFLICT OF INTEREST

None declare.

## FUNDING INFORMATION

None.

## Data Availability

The datasets generated during and/or analysed during the present study are available from the corresponding author on reasonable request. Data sharing is not applicable to this article because no data sets were generated or analysed during the present study. The data cited are all contained in the article by Roach et al. (2023), *Exp Brain Res*, *241*: 2433–2450. https://doi.org/10.1007/s00221‐023‐06689‐4.
